# Biology, Control and Zoonotic Role of Disease Vectors

**DOI:** 10.3390/pathogens12060797

**Published:** 2023-06-02

**Authors:** Rodrigo Morchón, Rubén Bueno-Marí, Daniel Bravo-Barriga

**Affiliations:** 1 Zoonotic Diseases and One Health Group, IBSAL-CIETUS (Biomedical Research Institute of Salamanca-Research Centre for Tropical Diseases), Faculty of Pharmacy, University of Salamanca, 37007 Salamanca, Spain; 2 Center of Excellence in Vector Control for Europe, Rentokil Initial, 46960 Valencia, Spain; ruben.bueno@uv.es; 3 Parasites & Health Research Group, Department of Pharmacy, Pharmaceutical Technology and Parasitology, Faculty of Pharmacy, University of Valencia, 46100 Burjassot, Valencia, Spain; 4 Parasitology and Parasitic Diseases, Animal Health Department, Veterinary Faculty, University of Extremadura, 10003 Cáceres, Spain; dbravoparasit@unex.es; 5 Department of Animal Health, Animal Health and Zoonosis Research Group (GISAZ), UIC Zoonosis and Emerging Diseases (ENZOEM), University of Córdoba, 14014 Córdoba, Spain

Vector-borne diseases result from the transmission of pathogens, including bacteria, parasites or viruses, by different hematophagous insects such as mosquitoes, phlebotomine sandflies, black flies, ticks, fleas, lice and triatomines, among others [1]. These vectors can transmit infectious pathogens between humans, animals, and vice versa throughout their lifetime with each bite or subsequent ingestion of blood [2].

These diseases account for over 17% of all global infectious diseases and cause more than 700,000 deaths per year [3]. Vector control is the primary method of combating these diseases, as there are often no alternative drug treatments or vaccines available [4]. Another approach is addressing the interactions between pathogens, hosts and the environment, which are crucial in understanding the emergence or re-emergence of these diseases [5].

Culicid mosquitoes are the most significant biological vectors in terms of morbidity and mortality associated with vector-borne diseases. Globally, there are an estimated 219 million cases of malaria transmitted by anopheline mosquitoes, causing more than 400,000 deaths per year. *Aedes* mosquitoes are responsible for the transmission of dengue, the most prevalent viral infection, along with other arboviruses such as Zika, Chikungunya and Mayaro, which have spread in recent years. The situation with West Nile virus (mainly linked to *Culex* mosquitoes) is similar, and this zoonosis is now considered endemic not only in the original foci in Africa, but also in other continents such as Europe and the Americas [3]. Also noteworthy is the presence of sandflies and ticks, both of which are implicated in the transmission of diseases that significantly impact humans. Among the most important diseases associated with them are leishmaniosis and phlebovirus infections, as well as Lyme encephalitis/disease and Crimean–Congo hemorrhagic fever, respectively.

The transmission of a wide range of vector-borne diseases is being influenced by climate change as vectors move to latitudes and altitudes higher than those that were previously reported [6].

The aim of this Special Issue was to bring together various studies about the presence of vectors, the spread and causes of vector-borne diseases, and vector–pathogen interactions including bionomic studies, surveillance projects and control experiences, among others. A study conducted by Wang et al. [7] found that high doses of X-rays could be used to reduce the sterility of *Ae. albopictus* males in a laboratory setting. However, they had no effect on egg numbers but did significantly reduce the survival time and hatching rate. Claver et al. [8] reported relatively modest vector competence for *Aedes aegypti* in Vientiane, Lao PDR, to transmit the Asian and ECSA-IOL lineages of chikungunya virus, which may be influenced by the longevity and density of female mosquitoes. Leandro et al. [9] evaluated the spatial and temporal association between vector infestation and the occurrence of dengue cases and reported a dynamic pattern indicating significant risks in certain areas of Foz do Iguaçu, Brazil, based on the entomological–virological index, while entomological indices were not effective in measuring dengue risk. Another study tested the efficacy of modifications to sewerage structures as an alternative to the use of biocides to prevent the breeding of *Culex pipiens* and *Aedes albopictus* in Barcelona, Spain [10]. Placing a concrete layer on the bottom of the sand sewers to prevent water accumulation completely eliminated mosquito breeding and, therefore, the need for biocides in the modified structures. Moreno-Gómez et al. [11] evaluated the effectiveness of the insecticide pyrethroid transfluthrin in providing protection against *A. albopictus* bites in humans, reporting high protection with mosquito mortality declining rapidly. In their comprehensive review, Morchón et al. [12] shed light on the epidemiological landscape of heartworm disease in Europe, uncovering its alarming spread and establishment as an endemic condition. The authors discuss a diverse array of dirofilariosis vectors and highlight key factors fueling this expansion, such as climate change, the emergence of new vectors, pet mobility, urbanization and the proliferation of irrigated crop regions. González et al. [13] providing new records for the Dominican catalogue of Diptera (*Culex salinarius* for the Greater Antilles, *Culicoides jamaicensis* for Hispaniola, and *Culicoides haitiensis* and *Culicoides borinqueni* for the Dominican Republic) consisting of the first COI DNA sequences available from different Diptera in GenBank. They discussed the spatial distribution, feeding preferences, and diagnostic features of closely related specimens in the Caribbean region. Guillot et al. [14] demonstrated that active sentinel surveillance of nymphal ticks provides a sustainable system for tracking enzootic risk for Lyme disease in southern Quebec. Lastly, Alevi et al. [15] presented the state-of-the-art taxonomy of the entire subfamily Triatominae, highlighting the transition from classical studies to the use of integrative taxonomy.
